# Caprine Paratuberculosis Seroprevalence and Immune Response to Anti-*Mycobacterium avium* Subspecies *paratuberculosis* Vaccination on the Canary Islands, Spain

**DOI:** 10.3390/vetsci11090388

**Published:** 2024-08-23

**Authors:** Elena Plamenova Stefanova, Yania Paz-Sánchez, Óscar Quesada-Canales, María del Pino Quintana-Montesdeoca, Antonio Espinosa de los Monteros, Ana Sofía Ramírez, Antonio Fernández, Marisa Andrada

**Affiliations:** 1 Veterinary Histology and Pathology, Institute of Animal Health and Food Safety (IUSA), Veterinary School, University of Las Palmas de Gran Canaria, 35413 Arucas, Gran Canaria, Spain; oscar.quesada@ulpgc.es (Ó.Q.-C.); antonio.espinosa@ulpgc.es (A.E.d.l.M.); antonio.fernandez@ulpgc.es (A.F.); marisaana.andrada@ulpgc.es (M.A.); 2 Departament of Morphology, Veterinary School, University of Las Palmas de Gran Canaria, 35413 Arucas, Gran Canaria, Spain; 3 Departament of Mathematics, University of Las Palmas de Gran Canaria, 35017 Las Palmas de Gran Canaria, Gran Canaria, Spain; mariadelpino.quintana@ulpgc.es; 4 Epidemiology and Preventive Medicine Unit, Institute of Animal Health and Food Safety (IUSA), Veterinary School, University of Las Palmas de Gran Canaria, 35413 Arucas, Gran Canaria, Spain; anasofia.ramirez@ulpgc.es

**Keywords:** ELISA, Johne’s disease, goat, antibody, serological test, age, mycobacteria

## Abstract

**Simple Summary:**

Paratuberculosis (PTB) is a chronic disease that affects domestic and wild ruminants worldwide. This study was conducted in 12 diary caprine farms on the Canary Islands. The region counts with the fourth largest goat population in Spain and has “officially free” bovine tuberculosis status. Two sampling sessions were conducted, and 2774 serum samples were tested by an enzyme-linked immunosorbent assay. In the first session, a prevalence of 18.4% was obtained, varying from 2.5% up to 61.1%. In the second session, the effect of PTB vaccination was evaluated and both non-vaccinated (nV) and vaccinated (V) were included. Variable tendencies in antibody development were registered in farms with different initial seroprevalences. In farms in which up to 10% of the animals were positive, more adult goats had antibodies against PTB after vaccination. In farms with more than 10% of ELISA-positive animals, a heterogeneous response to vaccination was reported. We observed that in farms with higher initial prevalence, fewer goats that were V developed antibodies. Our work characterizes the caprine PTB situation on the Canary Islands and gives new insights on the effect of farm prevalence on the immune response to PTB vaccination, although further studies on a greater scale are needed.

**Abstract:**

Paratuberculosis (PTB), caused by *Mycobacterium avium* subspecies *paratuberculosis* (MAP), is a chronic disease with economic impact on ruminant farming worldwide. The Canary Islands count with the fourth largest goat population in Spain and are “officially free” of bovine tuberculosis. Twelve farms were included with 2774 serum samples tested by an enzyme-linked immunosorbent assay (ELISA) for detection of anti-MAP antibodies in two sessions. In the first session, an overall apparent prevalence of 18.4% (2.5% up to 61.1%) was obtained. Farms with prevalences (0–10%], (10–20%] and >20% were identified, with differences in seroconversion in the same prevalence group between farms and age ranges. Non-vaccinated (nV) and vaccinated (V) animals were included in the second sampling session. Higher levels of antibodies were detected in V animals older than 12 months, with considerable variations between age ranges and farms. Our results describe the current PTB status of the Canary Islands’ goat farming. Furthermore, new insights on the effect of the farm prevalence on seroconversion in V animals are provided, although further studies are needed to evaluate the multiple factors affecting the immune response to anti-MAP vaccination.

## 1. Introduction

Paratuberculosis (PTB), also known as Johne´s disease, is a chronic wasting disease caused by *Mycobacterium avium* subspecies *paratuberculosis* (MAP) that affects both domestic and wild ruminants worldwide [1,2,3,4,5,6]. Animals are usually infected at a young age by ingestion of fecal material present in a contaminated environment, water, or food, as well as by drinking milk or colostrum from infected adult animals [3,4,6]. Intrauterine infection has also been described [1,3,4,6].

Although animals get infected at a young age, the subclinical phase is long, and clinical signs such as watery diarrhea, weight loss, and reduction in milk production appear only in the terminal phase [7,8]. Furthermore, the clinical signs in small ruminants are not as straightforward as in cattle [3]. In goats, data about the effect of age on clinical signs development are limited. Nevertheless, infected ruminants can eliminate MAP in their feces from initial stages of infection, which makes early diagnosis very important for correct control and prevention of the disease in affected areas [3,4,5].

PTB is a World Organization for Animal Health (WOAH)-listed disease and must be reported to this organization as indicated in the Terrestrial Animal Health Code [9]. Furthermore, according to the renewed animal health European Union (EU) legislation (Regulation (EU) 2018/1882), PTB is listed as a ‘category E disease’ for which there is a need for surveillance within the Union, as referred to in Article 9(1)(e) of Regulation (EU) 2016/429. The definitive confirmation method includes post-mortem identification of PTB-compatible lesions and histopathological examination [1,2,3,10]. Nevertheless, early ante-mortem diagnosis is hampered by the relatively low sensitivity of diagnostic tests on an individual level and the lack of pathognomonic signs, which leads to well-established infection in the herd before the first case is diagnosed [3,4,6,11]. 

Control programs usually include immunodiagnostic tests such as the enzyme-linked immunosorbent assay (ELISA), agar gel immunodiffusion (AGID) assay, intradermal skin testing, lymphocyte transformation, and IFNγ assays [3,5,12]. Polymerase chain reaction (PCR) targeting the insertion sequence 900 (IS 900) in fecal samples is a frequently used technique for herd screening as it has proven to be one of the most sensitive methods for MAP DNA detection. Microbiological culture, on the other hand, is a gold standard for PTB confirmation, although it is timely, and its detection capacity is limited in the early stages of PTB [12,13].

Furthermore, vaccination is used as a control tool as it has proven to reduce but not eliminate MAP fecal shedding, clinical cases, and lesions in target organs of affected animals [11,12,14,15,16]. Nevertheless, anti-MAP vaccination is banned in some countries, such as Denmark, and requires a special authorization in France, Germany, and Spain [12]. Only some countries apply PTB vaccination on goats, including Australia, Spain, and the Netherlands [12]. In Spain, vaccination in some regions, such as the Canary Islands, is subject to special authorization from the local authorities due to its interference with tuberculosis (TB) diagnosis. Although data about the seroconversion of vaccinated goats is scarce, some authors suggest that it might be related to various factors, including the age of vaccination and the environmental MAP dose to which animals are exposed on the farm [17,18]. 

However, the use of anti-MAP vaccines is controversial as it has been proven to interfere in the interpretation of intradermal skin tests, which are used in eradication programs of mycobacterial infections such as bovine tuberculosis (TB) [11,12,16].

In recent years, goat production has increased worldwide, mainly in developing countries, due to the low input it requires [5,6,19]. The small ruminant industry has significantly contributed to the alleviation of poverty in Africa and Asia [5]. Currently, Europe bares the third-largest goat production [20]. Spain has the second largest population on the continent, with a total of 2.293 million heads (2023) [21], distributed mainly between 5 autonomous communities. Andalucía counts with 37% of the population, Castilla-La Mancha with 15%, Extremadura with 10%, the Canary Islands with 10.3%, and Murcia with 9.7% [20]. Data about PTB prevalence, however, are limited, except for Andalucia, where a recent study established a seroprevalence of 20% [4]. Nevertheless, the disease is considered widespread in continental Spain, originating considerable economic losses [4,22]. 

The present study was conducted on the Canary Islands, which counts with the fourth largest Spanish goat population with a total of 200,054 heads and 1256 farms [23] of mainly certified autochthonous endangered breeds (Orden APM/26/2018). Most of the goat population is centered on the islands of Fuerteventura and Gran Canaria, which count with 73,572 and 47,388 heads, respectively [23]. Since the region was granted an “officially free” status for TB in 2017, vaccination against PTB is subject to a special protocol established by the local authorities (Decreto 51/2018 del 23 de abril) [24]. The aim of our work is to evaluate the current status of PTB on the Canary Islands throughout ELISA-based seropositivity measurement and the effect of vaccination on the humoral immune response in naturally infected herds.

## 2. Materials and Methods

### 2.1. Study Design and Sampling

#### 2.1.1. Animals and Farms

A total of 12 dairy caprine farms (5 from Fuerteventura and 7 from Gran Canaria) were sampled between 2018 and 2022. All animals were Majorera goats, a local certified autochthonous endangered breed (Orden APM/26/2018). All samples were submitted to the Institute of Animal Health and Food Safety (IUSA), Veterinary School, University of Las Palmas de Gran Canaria, as part of the official request process for an anti-MAP vaccination permit. In all farms, the presence of PTB was suspected by the identification of clinical signs, including severe emaciation, protrusion of lumbar vertebrae, easily palpable transverse processes, muscle mass loss, and a reduction in visceral fat deposits. 

The sampling was conducted in 2 sessions, and a total of 2774 serum samples were analyzed, sampling with a minimum expected prevalence of 10% and a 95% CI. In the first sampling session, approximately 15% of the census of each farm was tested. Subsequently, 7 farms (3 from Fuerteventura and 4 from Gran Canaria) were granted an authorization for vaccination against PTB. A second sampling session was conducted 12 months after the first session in 9/12 farms, and 1274 serum samples were analyzed. Both vaccinated (V) and non-vaccinated (nV) animals were included in the second sampling session as the local legislation specifies that a control group of nV animals should be left on farms that are granted a vaccination permit (Artículo 4 del Decreto 51/2018 del 23 de abril) [24]. The number of serum samples per farm and sampling session is summarized in Table 1.

#### 2.1.2. “TB-Free” Status Confirmation

This study was conducted on the Canary Islands, in which PTB vaccination is regulated by the Decreto 51/2018 del 23 de abril [24], which marks the requirements to obtain a vaccination permit. Since the islands are “officially free” of bovine tuberculosis, farmers who wish to implement anti-MAP vaccination need to certify that the animals are free of tuberculosis and that PTB is present in the farm [25,26]. As part of this process, all farms that requested vaccination were subjected to an on-field comparative intradermal tuberculin (CIT) test for detection of the *Mycobacterium tuberculosis* complex. All animals with positive or inconclusive results were send to slaughter. The absence of tuberculosis in those was confirmed by histopathology and bacterial cultures performed by the laboratory of VISAVET, Health Surveillance Centre, Madrid, Spain. All farms included certified that they were officially TB-free. 

#### 2.1.3. PTB Confirmation on Herd Level

In all farms included in this study, PTB was confirmed on herd level by post-mortem examination by necropsy performance and/or sampling at slaughter in all farms [25,26]. Afterwards, histopathological identification of granulomatous lesions affecting the mesenteric lymph nodes and/or the ileocecal valve was performed, as well as Ziehl–Neelsen for identification of acid-fast bacteria and/or immunohistochemistry for identification of MAP antigens [25]. In 3 farms, an additional real-time polymerase chain reaction (PCR) for MAP DNA detection targeting the insertion sequence 900 (IS900) was performed on tissue samples [25]. Details about the PTB confirmation techniques used in each farm are summarized in Table 1.

### 2.2. Serum Sampling

The whole blood samples were obtained by puncture of the jugular vein using sterile tubes without anticoagulant (Vacutainer^®^, Becton-Dickinson, Franklin Lakes, NJ, USA). Subsequently, those were transferred to the laboratory under refrigeration within the first 24 h after the sampling. Afterwards, the blood was centrifuged at 400 g for 10 min, and serum was obtained. Samples were stored at −20 °C until the analysis was performed.

### 2.3. Anti-MAP Vaccine

The anti-MAP vaccine applied was Gudair^®^ commercial heat-inactivated vaccine containing 2.5 mg/mL of MAP strain 316 F with mineral oil adjuvant (CZ Vaccines S.A., O Porriño, Pontevedra, Spain) for use in sheep and goats. One milliliter of vaccine was subcutaneously administered in the post-scapular area of the back following the manufacturer’s instructions and the guidelines of the Spanish Agency for Medicines and Medical Devices (AEMPS), which indicate that in heavily affected herds, all animals, including adult ones, should be vaccinated. In the herds that were granted vaccination permission from the local authorities, a control group of nV animals was left as required by the local legislation (Decreto 51/2018 del 23 de abril) [24]. These goats were managed under the same conditions as the V ones.

### 2.4. Serological Assay

Serum samples were analyzed using a commercial in vitro diagnostic ELISA test kit for detection of antibodies to *Mycobacterium avium* subspecies *paratuberculosis* (*Mycobacterium paratuberculosis* Test Kit PARACHEK^®^ 2, Prionics AG, Schlieren, Switzerland) following the manufacturer’s protocol. According to the data provided in the data sheet, the sensitivity (Se) in goats ranges from 65 to 88%, and the specificity (Sp) is of 99% or greater. 

### 2.5. Farm Characterization

An official geographic tool designed by the local authorities on the Canary Islands (Sistema de Información Territorial de Canarias^©^ GRAFCAN 1989-2024) was used to collect data on the production characteristics of the farms. Information about the following general production and biosecurity variables was extracted for each farm: altitude, distance to a main road, presence of other farms in a perimeter of 1 km^2^, livestock perimeter fencing, and mean annual temperature. 

### 2.6. Prevalence Groups

Farms were categorized in the following groups based on the within-herd apparent prevalence: group A, farms with (0–10%] seropositivity; group B, (10–20%] seropositivity; and group C, with ≥20% seropositivity.

### 2.7. Statistical Analysis

An observational cross-section study was conducted. The apparent seroprevalence was calculated separately on farm level. The true seroprevalence was calculated using the Se and Sp of the kit used, using the lowest Se value indicated by the manufacturer (65%).

Statistical analysis of data was performed by IBM SPSS Statistics 27 (IBM SPSS Statistics for Windows, Version 27.0. Armonk, NY, USA: IBM Corp). The age was summarized using the mean, standard deviation (SD), median, and interquartile range (IQR). Shapiro–Wilk and Kolmogorov–Smirnov tests were used to analyze the age and mean annual temperature normality. A non-parametric Mann–Whitney U and Kruskal–Wallis tests were used to compare the means of two independent samples (age/mean annual temperature and ELISA results). Categorical variables were summarized using percentages and relative or absolute frequencies. The ages of the studied goats were categorized in the following groups: (0–12) months, [12–24) months, [24–36) months, [36–48) months, [48–60) months, and ≥60 months. A chi-square test was used to contrast the association between two categorical variables. Additionally, the Bonferroni correction for multiple testing was applied to control experiment-wise and family-wise error rates.

The results were considered statistically significant if the *p*-value < 0.05.

## 3. Results

### 3.1. First Sampling Session

#### 3.1.1. Seroprevalence

The overall apparent individual seroprevalence was 18.4% (257/1500). The within-herd seroprevalence varied from 2.5% up to 61.1%, and this difference was statistically significant between the farms (*p* = 0.001). In all farms, positive animals were detected. The Se and Sp were used to calculate the true prevalence. The individual true prevalence was 27.19%, and the within-herd antibody detection ranged from 2.34% to 93.92%.

Subsequently, three prevalence groups were established for further analyses: group A with farms with (0–10%]; group B with (10–20%], and group C with >20%. In group A, 5 farms with a total of 379 animals were included. In group B, 3 farms with 256 animals were sampled. In group C, 4 farms with 865 goats were evaluated. The farms within-herd seroprevalence of each farm are summarized in Table 2. 

#### 3.1.2. Age Analysis

The animals sampled in the first session were between 1 and 210 months old, with a mean of 20.44 months, a median of 12 months, a SD of 17.03 months, and an IQR of 12 months. The goats were included in the following age groups: 128 had (0–12) months, 962 had [12–24) months, 172 had [24–36) months, 102 had [36–48) months, 81 had [48–60) months, and 44 had ≥60 months of age. No information was available about the age of 11 goats. Thus, 64.6% of the studied animals had between [12 and 24) months (19.9% positive and 80.1% negative).

**Figure 1:**
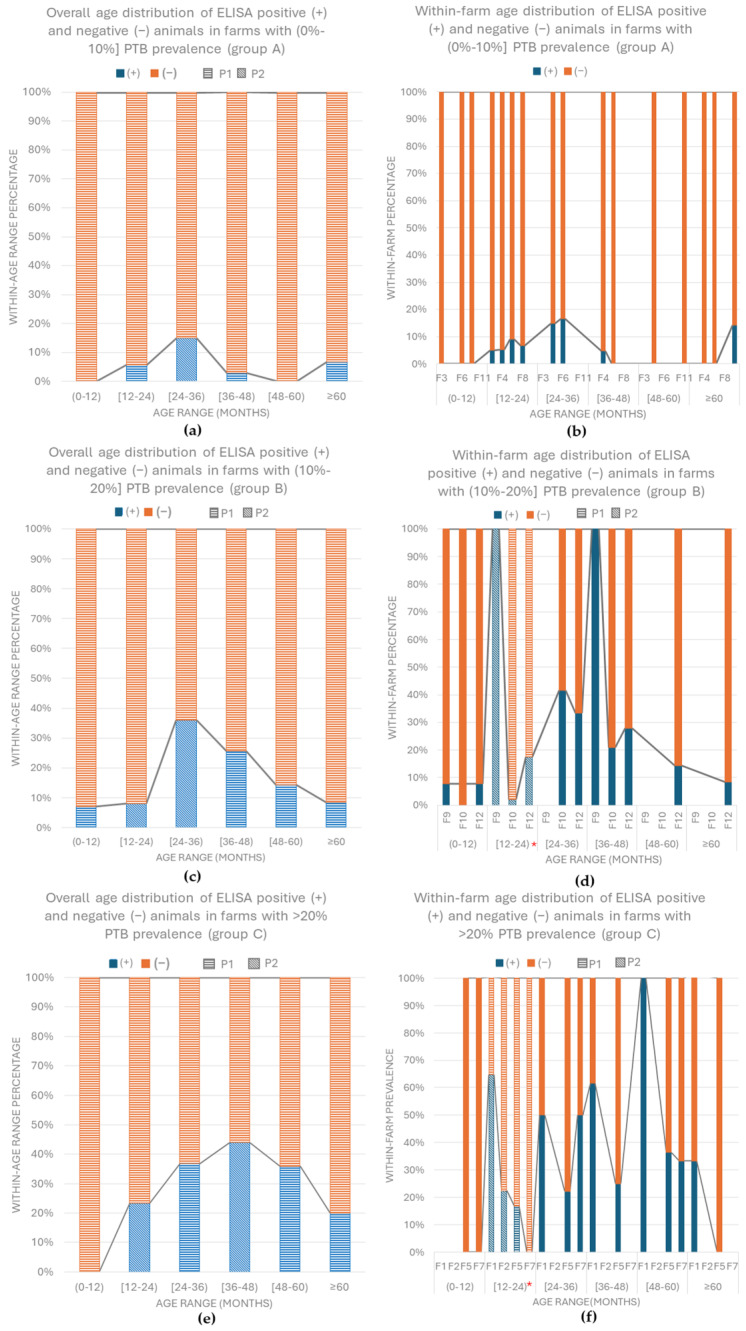
Overall and within-farm seroprevalence of the first sampling session per age range and prevalence group. Each bar pattern (P1 and P2) denotes a subset of within-farm ELISA result categories in the same age range whose column proportions do not differ significantly from each other at the 0.05 level (Bonferroni correction). (**a**) Overall seroprevalence in farms from group A (0–10%] per age group; (**b**) within-farm seroprevalence in farms from group A (0–10%]; (**c**) overall seroprevalence in farms from group B (10–20%] per age group; (**d**) within-farm seroprevalence in farms from group B (10–20%]. Statistical differences were demonstrated between farms in the age group [12–24) months, * (*p* = 0.001). (**e**) Overall seroprevalence in farms from group C (>20%) per age group; (**f**) within-farm seroprevalence in farms from group C (>20%). Statistical differences were demonstrated between farms in the age groups [12–24) months * (*p* = 0.001).

In group A, no statistical differences were present between the within-farm ELISA results regardless the age of the animals (*p* = 0.200) (Table 3). Nevertheless, statistical differences were demonstrated between the ELISA results in the different age ranges (*p* = 0.002) with proportional differences in the group of (0–12) with 0% of positive animals and [24–36) with 14.92% of positive goats (Figure 1a). Within each age range, no differences were detected between farms. Furthermore, the proportions in each age range were separately assessed and no differences were demonstrated (Figure 1b). 

In group B, the differences between the farms, regardless of the age, were not significant (*p* = 0.549) (Table 3). Nevertheless, statistical differences were demonstrated between the results in the different age ranges (*p* = 0.001) with proportional differences in the animals from [12–24) and [24–36) months of age with 8% and 35.9% of positive goats, respectively (Figure 1c). Once the results in the different age ranges were compared between the farms, differences were demonstrated in the predominant age group of [12–24) months (*p* = 0.001) in which the ELISA-positive animals in the three farms were 100%, 2%, 17.4%, respectively. However, no proportional differences were detected (Figure 1d). 

In group C, statistical differences between the four farms in relation to the number of positive and negative animals were demonstrated regardless of the age of the animals (*p* = 0.001) (Table 3). Once the age of the animals was assessed without taking into account the farm of origin, the differences were also significant (*p* = 0.014), with proportional differences being present in the groups of (0–12), [12–24), and [26–48) months with 0%, 23.3%, and 44% of seropositivity, respectively (Figure 1e). Furthermore, association between the within-farm results was also confirmed in the age group of [12–24) months (*p* = 0.001), in which farms the seroprevalences were of 0%, 16.7%, 22.4%, and 64.7%, with a significant proportional difference being present (Figure 1f). 

#### 3.1.3. Farm Characterization

The farm characterization (Table 2) carried out demonstrated that the majority of the herds included in this study were completely fenced, with only 2/12 being not fenced. Statistical differences between ELISA results and the presence of fencing were not significant in neither group A, in which all farms were fenced, nor in group B (*p* = 0.298). In group C, 61.1% of the animals from non-fenced farms were positive in contrast with 22.8% from farms with complete fencing and the differences were significant (*p* = 0.001). The average altitude was of <600 m, with 2/12 farms being situated on higher altitudes. No statistical differences between the ELISA results and the altitude of the farms were detected in group A, in which all farms were situated on <600 m, group B (*p* = 0.597), or group C (*p* = 0.360). The mean annual temperature ranged from 15 °C to 21.40 °C with 2/12 farms presenting less than 19 °C. Significant associations between the ELISA results and the mean annual temperature were not detected, neither in group A (*p* = 0.062) nor group B (*p* = 0.597). In group C, F1 and F7 presented the highest seroprevalences and the lowest mean annual temperatures, and the differences were significant (*p* = 0.001). Regarding the distance to a main road, only 2/12 farms was situated on >1 km from the closest main road. The differences between the ELISA results and the distance to the main road were not significant in group A (*p* = 0.075), group B (*p* = 0.448), or group C (*p* = 0.612). Lastly, only 3/12 farms did not have any other ruminant farm in the surrounding area. The ELISA result had no significant association with the surrounding farms in group A (*p* = 0.064) or group B (*p* = 0.597). In group C, 21.7% of positivity was registered in herds with no other farms in the surrounding area and 24.2% in herds with other ruminant farms present in the surroundings, and the difference was significant (*p* = 0.001). 

### 3.2. Second Sampling Session

A total of 1274 samples from 9/12 farms were tested 12 months after the first sampling session. Overall, a total of 35.1% (455/1274) of the animals tested positive. In the V group, 63.8% (233/365) goats tested positive, and in the nV, 24.4% (222/909).

#### Age Analysis

The age of the animals in the second sampling session ranged from 6 to 213 months, with a mean of 21.67 months, a median of 12 months, a SD of 21.13 months, and an IQR of 14 months. The number of goats in each age group was as follows: 409 had (6–12) months, 513 had [12–24) months, 143 had [24–36) months, 71 had [36–48) months, 39 had [48–60) months, and 99 had ≥60 months of age. Thus, 72.4% of the studied animals were between 6 and 24 months of age, of which 29.2% tested positive (72.9% nV and 27.1% V) and 70.8% were negative (89.3% nV and 10.7% V).

Furthermore, the differences between the positive and negative samples were analyzed separately in the V and nV animals in each prevalence group.

**Figure 2:**
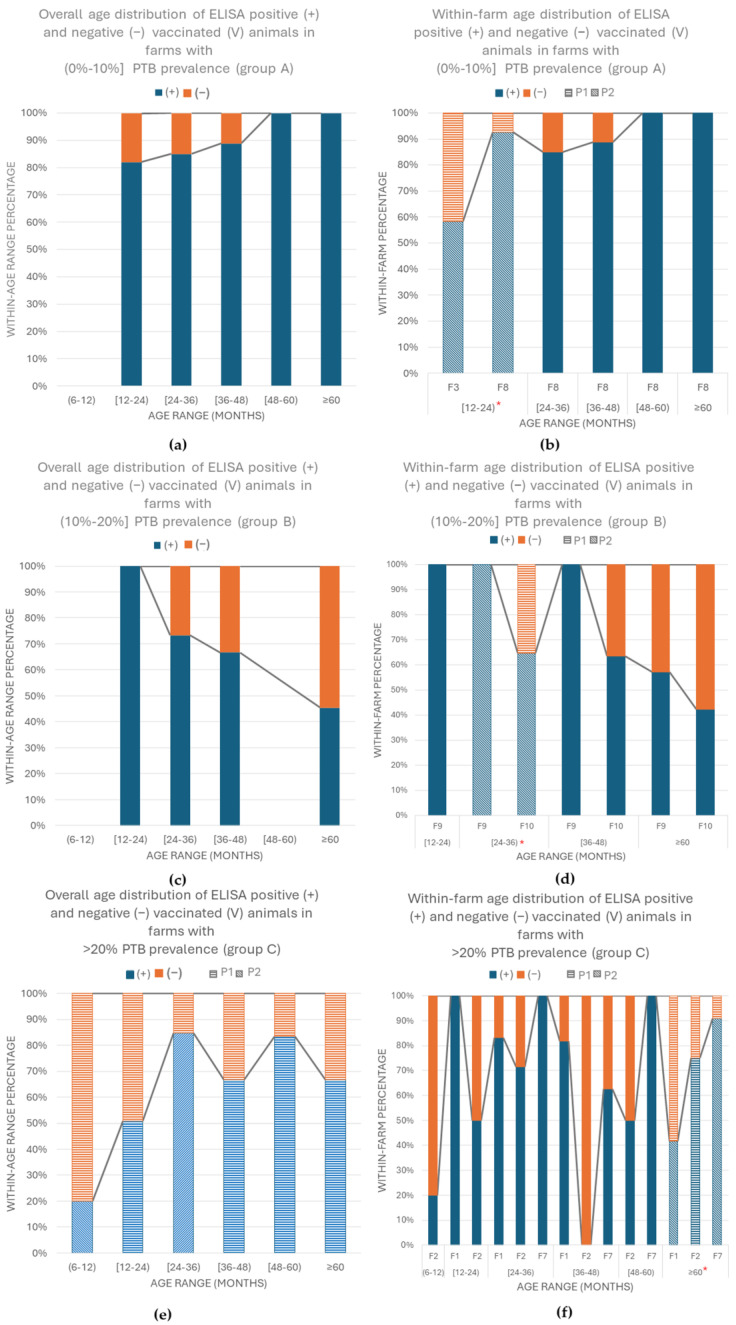
Overall and within-farm seroprevalence of the second sampling session per age range and prevalence group in anti-MAP-vaccinated (V) goats. Each bar pattern (P1 and P2) denotes a subset of within-farm ELISA result categories in the same age range whose column proportions do not differ significantly from each other at the 0.05 level (Bonferroni correction). (**a**) Overall seroconversion in V animals from group A (0–10%] farms per age range with a tendency of higher antibody levels in adult goats; (**b**) within-farm seroprevalence in V animals from farms in prevalence group A (0–10%] with statistical differences between farms in age group [12–24) months * *p* = 0.010 and higher percentage of seroconversion in older animals; (**c**) overall seroconversion in V animals from group B (10–20%] farms per age range with a tendency of higher antibody levels in younger goats; (**d**) within-farm seroprevalence in V animals from farms in prevalence group B (10–20%] with statistical differences between farms in the age group of [24–36) months * *p* = 0.021 and a general heterogeneous anti-MAP vaccination response in the different farms; (**e**) overall seroconversion in V animals from group C (>20%) farms per age range with proportional differences and lowest seroconversion in animals (6–12) months old and higher in [24–36) old goats; (**f**) within-farm seroprevalence in V animals from farms in prevalence group C (>20%). Statistical differences were demonstrated between animals ≥ 60 months, * *p* = 0.041, and a heterogeneous immune response to anti-MAP vaccination between farms.

In group A, a total of 195 animals from 2/5 of the originally sampled farms were included. A difference between the number of positive and negative animals in the farms was demonstrated in both V (*p* = 0.007) and nV (*p* = 0.001) goats, regardless of the age range (Table 3). In the case of the nV animals, this difference was confirmed in the age groups of (6–12) and [12–24) months in which one of the farms had no positive animals and in the other one 40% (*p* = 0.001) and 55.3% (*p* = 0.035) of the goats presented anti-MAP antibodies, respectively. Once the proportions in each age range were separately assessed, no differences were demonstrated. In the case of the V animals, no statistical differences were demonstrated between the ELISA results in the different age ranges (*p* = 0.928), although a higher percentage of positive animals was detected in older animals (Figure 2a). It is worth highlighting that no animals 6–12 months old were present in the farms from this group, and thus the immune response in young goats could not be evaluated. On the other hand, within each age range, differences between the farms were detected in the predominant age group of [12–24) months in which the 92.6% of the V goats from the first farm tested positive in contrast with 58.3% in the second farm (*p* = 0.010), although no proportional differences were detected (Figure 2b). No statistical analysis was performed in the other age groups, as only one farm had animals older than 24 months, but a tendency was observed of a higher percentage of seroconversion in older animals.

In the farms from group B, the same three farms included in the first sampling were checked, with a total of 183 sera obtained. Differences between the number of positive and negative goats were detected between both V (*p* = 0.019) and nV (*p* = 0.001) animals, regardless of the age range (Table 3). Regarding the nV animals, differences were present in the age range of [12–24) months in which the three farms presented a seroprevalence of 16.7%, 20%, and 76%, respectively (*p* = 0.002). Nevertheless, no proportional differences were demonstrated between the farms in this age range. In the case of the V goats, no statistical differences were demonstrated between the ELISA results in the different age ranges (*p*= 0.071), although seropositivity tended to decrease in older animals (Figure 2c). It is worth mentioning that no animals 6–12 months old were present in the farms from this group, and thus the immune response in young goats could not be evaluated. On the other hand, differences between the within-farms’ seropositivity were only demonstrated in the age group of [24–36) months in which one of the farms had only positive V animals in contrast with the other one in which only 64.7% of the goats presented anti-MAP antibodies (*p* = 0.021). The proportions, on the other hand, did not significantly differ (Figure 2d). The third farm did not implement vaccination as a control tool and thus had no V goats. In general, the seroconversion among the V animals from different farms was heterogeneous, being the lowest percentage of animals with anti-MAP antibodies from F10, in which the highest percentage of nV-positive animals was detected. 

Finally, in group C, all four originally tested farms were checked with a total of 896 samples. In one of them, vaccination was not implemented. A difference between the number of positive and negative samples in the different farms was demonstrated in the group of the V (*p* = 0.001) and the nV (*p* = 0.001) animals regardless of the age of the animals (Table 3). Regarding the nV animals, differences were demonstrated in the age range (6–12) and [12–24) months with *p* = 0.001 and *p* = 0.013, respectively. In the first age group, only F2 of the farms had positive goats (24.3%), and this proportional difference was statistically significant in comparison with F1 and F5, which had no positive animals. In the second age group, one of the farms presented 16.7% seropositivity in contrast with 22% and 58.3% in the other two, and the proportional difference was statistically significant at the 0.05 level. The last farm had no nV animals in this age group. Regarding the V goats, statistical differences were demonstrated between the ELISA results in the different age ranges (*p* = 0.001). The lowest seroconversion levels were registered in the animals (6–12) months old with 20% and the highest in the age range of [24–36) months with 80.2% of seropositivity (Figure 2e). Significant proportional differences were also confirmed at 0.05 level. Regarding the within-farm seropositivity, in the three farms in which vaccination was implemented, differences were confirmed only between the oldest animals (≥60 months), in which in one of the farms 75% of the V goats presented anti-MAP antibodies in contrast with 41.6% and 90% in the other two (*p* = 0.041). This proportional difference was statistically significant at the 0.05 level (Figure 2f). The farm with the lowest level of seroconversion in this age group was F1, which is the farm with the highest seroprevalence in the first sampling (61.1%). In general, the seroconversion, although not statistically significant, was heterogeneous among the different farms. 

## 4. Discussion

Paratuberculosis is a well-known problem in ruminant farms worldwide and is also a WOAH-listed disease and thus must be notified as marked by the Terrestrial Animal Health Code [9]. Nevertheless, a review published in 2019 suggested that in 74% of the countries in which PTB was notifiable, it was underreported [12]. In Spain, various studies have been published stating that PTB is a widespread problem [4,6,22]. Although, in the second half of 2022, only 224 cases were reported in ovine and caprine species in Spain and none of them in the Canary Islands [27]. 

Furthermore, PTB’s prevalence is considered to be underestimated regardless of the geographic location [4,12]. The main reasons for this situation included in a recent report are as follows: low sensitivity of the diagnostic test, lack of surveillance, and lack of knowledge or awareness on the clinical signs of the disease [12]. In Spain, various reports have been published in order to estimate the prevalence of the disease in different regions of the country [4,6,28]. To the authors’ knowledge, this is the first study reporting the current caprine PTB status of the Canary Island archipelago, which bears the fourth largest goat population in Spain [20]. The overall apparent individual seroprevalence of 18.4% found in our work is slightly lower than two previous descriptions conducted in the region of Andalucia, which carries the largest goat population in the country. These reports detected goat-positive serum samples in 20% and 22.5% [4,6]. It is worth mentioning that the sensitivity of the ELISA is relatively low, mainly in the early stages of the disease, and thus, as mentioned in other studies, the true PTB prevalence might be underestimated [3,4,6,12,29].

On the other hand, biosecurity measures are considered one of the most effective preventive strategies against PTB spread in a herd and between herds [3,4,5,7,8,30]. Although data about exact risk factors in goat farms is limited, a previous study conducted in Spain shows that the risk of seropositivity to MAP was 2.2 times higher in farms without full perimeter fencing [4]. In our work, the highest herd prevalence was found in a non-fenced farm with other ruminant farms nearby (F1, 61.1%). Furthermore, the statistical differences between the number of positive and negative animals in the farms from group C in relation to the fencing, the mean annual temperature, and the presence of other ruminant farms in the surrounding area suggest the important role of the farm characteristics and biosecurity measures on the animals’ anti-MAP immune response and PTB status [3,17]. 

Furthermore, the age of the affected animals was analyzed in detail, as it is a well-known fact that the incubation phase of PTB is long [3,5,12,13]. However, data about how long a goat can shed MAP and exhibit no clinical signs are scarce. In sheep, a review study stated that usually clinical PTB is detected in animals older than 2 years, with many being older than 4 years [31]. In cattle, cases of asymptomatic infected cows of up to 14 years have been described [31]. In our study, 64.6% of the studied animals had between 12 and 24 months of age, which can be explained by the standard productive age range in caprine farming. Furthermore, the results of the present work show a tendency of higher immune response being detected in older animals, regardless of the prevalence group of the farms. In a recent study from a naturally infected farm with relatively low prevalence, Fernandez et al. detected higher initial antibody levels in the adult animals older than 1.5 years from the non-vaccinated control group in contrast with the animals younger than 6 months [15]. Another study from Mercier et al. in heavily infected farms detected an increase in the seropositivity of the control group of goat kids 15.5 months after the beginning of the study and even higher levels once 23 months had passed [32]. Furthermore, in all prevalence groups, animals older than ≥60 months were present, and a considerable part tested positive. Those animals could be potential shedders of the disease into the environment and, given the relatively low Se of the ELISA test, their number might be underestimated [4,5,6,33,34]. Thus, our results highlight the importance of future studies analyzing the role of keeping old animals in PTB-affected farms on the prevalence and dissemination of the disease in the herd. On the other hand, the statistical differences demonstrated in the main age groups from the included farms with similar prevalences confirm that, as suggested by other authors, anti-MAP antibody development is multifactorial and not strictly age-related. In naturally infected herds, doses and infection routes are different, and the disease stages of the included animals can vary and should be considered [3,5,31,34]. 

Lastly, this work evaluates the effect of age on seroconversion once anti-MAP vaccination is implemented. Numerous studies have demonstrated the beneficial effects of PTB vaccination on the reduction in clinical cases and histopathological lesions in affected herds [5,11,14,15,35]. Nevertheless, vaccination implementation does not prevent infection; although MAP shedding in vaccinated animals is reduced, those can still eliminate MAP into the environment and stay infectious [11,12,14,36]. Based on those facts and following the manufacturer’s instructions, in heavily infected herds, both young and adult animals were vaccinated. In our work, 63.8% of the V animals developed anti-MAP antibodies with variable seroconversion tendencies in the different prevalence groups. In group A, in which the farms had relatively low initial prevalence, a clear tendency of higher seropositivity in older animals was observed. Similar results were observed in group C, which was the only one in which animals between 6 and 12 months could be evaluated, and those were the ones with lower antibody levels. These results are in line with a study published by Corpa et al. that showed that the antibody response was higher in animals vaccinated at 5 months of age in contrast with those 15 days old [18]. The authors’ hypothesis states that the effect on antibody development might be explained by the fact that the immune system is mature in older animals in contrast with goat kids. In group B, however, the tendency was for a decrease in the antibody levels in older animals. Nevertheless, that tendency was not identic in the two farms evaluated. Furthermore, although it was not statistically demonstrated, farms with initial higher seroprevalences had fewer V ELISA-positive goats. A recent study suggested that the presence of anti-MAP antibodies in vaccinated animals might be related to the prevalence of environmental MAP in the farm they are raised in and is not strictly related to the age of vaccination [17]. Furthermore, in our work, the farm with higher seroprevalence (F1, 61.1%), presented the lowest percentage of animals ≥ 60 months of age that developed anti-MAP antibodies. Previous studies of mycobacterial infections, including the BCG vaccine in humans, have highlighted the possibility of elevated presence of mycobacteria in the environment to be the cause of blocking or masking the immune response in adult animals with matured immune system that have been continuously exposed to MAP [15]. 

Finally, it is worth highlighting that non-vaccinated animals were included in the second sampling, and although the levels of anti-MAP antibodies were similar, a seroprevalence of 24.4% was registered in contrast with the initial 18.4% in the first sampling. These results demonstrate the importance of serological surveillance in order to detect, assess, and control PTB in affected herds even after vaccination, as it does not prevent infection nor eliminate MAP shedding [5,12,31,37]. Nevertheless, ELISA tests, although widely used and cost-effective, still have limitations such as low sensitivity and the impossibility to differentiate between vaccinated and infected animals [12,13,16,17,18]. Thus, serological screening should be combined with other widely used MAP detection techniques, such as fecal PCR targeting IS 900, to better assess PTB herd status [12,13]. 

## 5. Conclusions

The present study describes the current epidemiological situation of PTB on the Canary Islands, which holds the fourth largest goat population in Spain. We demonstrate that PTB is endemic on the islands, with an average apparent prevalence of 18.4% varying from 2.5% up to 61.1% between the farms included. Furthermore, we conclude that the age of the animals might be related to anti-MAP antibody development, with higher seroprevalences being detected in animals older than 12 months. On the other hand, we demonstrate that age might not be the only factor affecting seroconversion, as considerable differences are observed between farms from different prevalence groups and with variable biosecurity and environmental characteristics, which suggest the possible role of the environment on seroconversion. Nevertheless, animals ≥ 60 months old are present in most of the farms, and a considerable percentage of them are ELISA-positive. Thus, we highlight the importance of test-and-cull practices implementation for reduction in possible MAP-shedders and correct assessment of the disease on farm level. This work also shows that the use of ELISA serological tests to diagnose PTB is a useful tool and should be implemented as part of the control protocol in the farms from the Canary archipelago. Moreover, we analyze the effect of vaccination, demonstrating the differences between the seroconversion in young and adult animals. We illustrate that in some cases, young animals do not seroconvert as expected after vaccination. Also, we observe that the amount of MAP present in the herd might be related to the level of antibodies developed after vaccination. Nevertheless, further studies are needed to explore the effect of the environmental component as well as other risk factors on the epidemiology and pathogenesis of caprine PTB.

## Figures and Tables

**Table 1:** Number of caprine serum samples per farm and island and PTB herd confirmation techniques.

Farm	1st Sampling	2nd Sampling			PTB Herd Confirmation
		V	nV	Total	
Fuerteventura	1098	165	769	934	
F1	36	36	34	70	+
F2	740	117	623	740	++
F3	80	12	68	80	++
F4	196	-	-	-	+
F5	46	0	44	44	+
Gran Canaria	402	200	140	340	
F6	46	-	-	-	+
F7	43	30	12	42	+
F8	38	79	36	115	+
F9	15	20	10	30	+
F10	91	71	25	96	+
F11	19	-	-	-	+
F12	150	0	57	57	++
Total	1500	365	909	1274	

F, farm; V, vaccinated; nV, non-vaccinated. + Includes post-mortem necropsy/slaughterhouse sampling with gross and/or histopathological granulomatous lesions in mesenteric lymph nodes and/or ileocecal valve and Ziehl–Neelsen and/or immunohistochemistry-positive samples. ++ Includes post-mortem PTB confirmation and PCR-positive tissue samples for IS900 identification.

**Table 2:** Within-farm apparent and true seroprevalence and farm characterization.

Farm	Apparent Seroprevalence (%)	True Seroprevalence (%)	1st Sampling Session (Month)	Fencing (Y/N)	Altitude (m)	Mean Annual Temperature (°C)	Distance to a Main Road (km)	Ruminant Farms in the Surrounding Area (Y/N)
Group A (0–10%]								
F3	2.5	2.3	May	Y	<600	21.2	<1	Y
F4	9.7	13.6	May	Y	<600	21.4	>1	N
F6	6.5	8.6	October	Y	<600	20.4	<1	Y
F8	2.6	2.6	October	Y	<600	20.6	<1	Y
F11	5.3	6.7	October	Y	<600	20.7	0	Y
Group B (10–20%]								
F9	20	29.7	October	Y	>600	15	0	N
F10	12.1	17.3	October	N	<600	20.1	0	Y
F12	16.7	24.5	March	Y	<600	20.2		Y
Group C > 20%								
F1	61.1	93.9	October	N *	<600	19.4 *	<1	Y *
F2	22.4	33.5	June	Y *	<600	21.1 *	<1	Y *
F5	21.7	32.4	January	Y *	<600	20.2 *	>1	N *

F, farm; Y, yes; N, no; m, meter; km, kilometer; °C, degree Celsius. * Statistically significant associations with *p* < 0.05.

**Table 3:** Statistical associations between within-farm ELISA results in caprine farms from the same prevalence group.

Farm	1st Sampling Session		2nd Sampling Session			
	ELISA + (%)	*p*-Value ^1^	V		nV	
			ELISA + (%)	*p*-Value ^1^	ELISA + (%)	*p*-Value ^1^
Group A (0–10%]						
F3	2/80; 2.5%	0.200	7/12; 58.3%	**0.007**	33/68; 48.5%	**0.001**
F4	19/196; 9.7%		-		-	
F6	3/46; 6.5%		-		-	
F8	1/38; 2.6%		70/79; 88.6%		0/36; 0%	
F11	1/19; 5.3%		-		-	
Group B (10–20%]						
F9	3/15; 20%	0.549	17/20; 85%	**0.019**	2/10; 20%	**0.001**
F10	11/91; 12.1%		40/71; 56.3%		19/25; 76%	
F12	25/150; 16.7%		-		5/57; 8.8%	
Group C > 20%						
F1	22/36; 61.1%	**0.001**	25/36; 69.4%	**0.001**	0/34; 0%	**0.001**
F2	166/740; 22.4%		48/117; 41%		149/623; 23.9%	
F5	10/46; 21.7%		-		7/44; 15.9%	
F7	13/43; 30.2%		26/30; 86.7%		7/12; 58.3%	

^1^ Chi-square test. The results were considered statistically significant if *p* < 0.05 (**bold values**).

## Data Availability

The data presented in this study are available on request from the corresponding author. The data are not publicly available due to privacy.
